# Organogels of FmocFF: Exploring the Solvent-Dependent Gelmorphic Behavior

**DOI:** 10.3390/gels10110749

**Published:** 2024-11-19

**Authors:** Basanta Saikia, Dong Chen, Yovan de Coene, Stijn Van Cleuvenbergen

**Affiliations:** 1 Department of Chemistry, Molecular Imaging and Photonics, KULAK—KU Leuven, E. Sabbelaan 53, 8500 Kortrijk, Belgium; bsaikia1@gmail.com (B.S.); dong.chen@kuleuven.be (D.C.); 2 Department of Chemistry, Molecular Imaging and Photonics, KU Leuven, Celestijnenlaan 200D, 3001 Heverlee, Belgium; yovan.decoene@kuleuven.be

**Keywords:** organogel, FmocFF, solvent effect, gel morphology, SHG microscopy

## Abstract

FmocFF (9-fluorenyl methoxycarbonyl-phenylalanine) is an extensively studied low-molecular-weight hydrogel. Although there have been numerous studies on FmocFF hydrogel, its potential to form organogels has not been well explored. In this work, we systematically explore the organogels of FmocFF in a wide range of organic solvents. FmocFF is found to be a robust organogeltor, and the subsequent organogels exhibit diverse gelmorphic behavior exhibiting various degrees of crystallinity and morphology depending on the solvent used. The mechanical strength of the organogels is evaluated using rheology. A novel technique, in situ SHG microscopy, is introduced to study the gel structure in its native state. In addition to the solvent–solute interactions that are typically used to predict gelmorphic behavior, we observed indications that the degree of crystallinity also plays a significant role in determining the mechanical properties and structure of FmocFF organogels.

## 1. Introduction

**Scheme 1:**
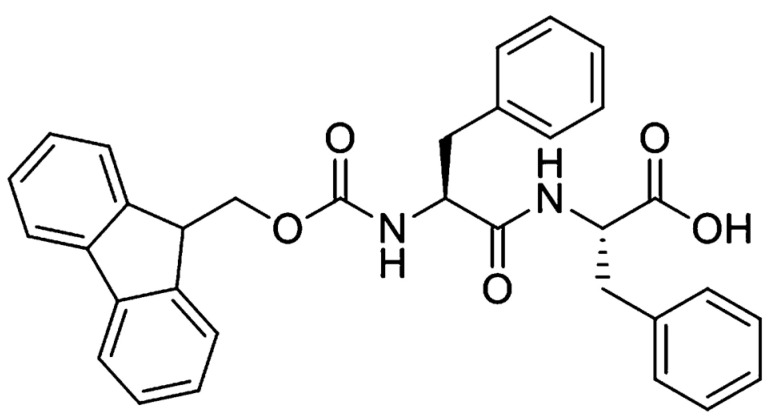
Chemical structure of FmocFF (9-fluorenyl methoxycarbonyl-phenylalanine).

Low-molecular-weight gelators (LMWGs) are a fascinating class of compounds which produce supramolecular gels via the self-assembly of gelator molecules (typically <2% by mass) into fibers creating a three-dimensional network that traps solvents to produce a viscoelastic material [1,2,3,4,5]. In LMWGs, the gelator molecules interact via noncovalent interactions like hydrogen bonding, π–π stacking, etc. These noncovalent interactions are significantly weaker than covalent bonds, typically ranging from 4–100 kJ/mol. The dynamic nature of different intermolecular interactions makes LMWGs highly responsive towards external stimuli such as temperature, solvent, pH, or concentration [6,7]. The formation of supramolecular aggregates in solution relies on a delicate balance of noncovalent interactions between solvent and gelator molecules. Unsurprisingly, the choice of solvent significantly influences the supramolecular gelation process, affecting everything from nucleation (the formation of an initial unstable nucleus) to the growth of gel fibers and the final structure of the gel network, which in turn impacts the gel’s mechanical properties [8,9,10,11,12]. To rationalize the effect of the solvent on gelation, researchers have sought to correlate quantitative solvent parameters to the emerging gel properties. In the case of hydrogen-bond-forming gelators, the Kamlet–Taft parameters, including solvent polarity and hydrogen bond donor/acceptor character, can provide a qualitative explanation of gel properties [9,12]. For instance, Smith et al. explored the role of Kamlet–Taft parameters of different solvents on the gelation ability of L-lysine bis-urea gelators with variable peripheral groups and concluded that in different solvents, the peripheral groups have different effects on gelation [9]. FmocFF (Scheme 1) is a low-molecular-weight gelator containing the aromatic fluorenylmethoxycarbonyl (Fmoc), with Fmoc-diphenylalanine, and has been extensively studied as a potent hydrogelator [13,14]. The mechanical properties of FmocFF hydrogels can be tuned by adjusting the experimental parameters like concentration, pH control, co-gelator addition, and overall gelation method [14,15,16]. This allows for the fabrication of hydrogels that closely mimic the physical characteristics of the target tissue, enhancing their suitability for applications such as cartilage and bone regeneration, wound healing, and drug delivery [17,18]. Though the hydrogel behavior of FmocFF is extensively studied, its organogels are relatively unexplored. Raeburn et al. studied FmocFF gel in mixed solvents, pointing out that solvent can change the microstructure of the fiber network in gel [15]. Understanding how a gelator like FmocFF behaves in organic solvents can open up new opportunities for using these gels in non-aqueous systems. Given the versatility of FmocFF due to its aromatic and hydrogen-bonding motifs, exploring its organogelation behavior could provide valuable insights into the structure–property relationships of organogels. Although organogelation is often serendipitous, FmocFF is a promising gelator considering the presence of its various terminal substituents. FmocFF molecules can be involved in carboxylic-to-carboxylic C(4) hydrogen-bonding motifs, such as 1D tapes via −C=O∙∙∙H−N that form R$\begin{array}{c} 2\\ 2 \end{array}$(10) synthons. Its propensity to aggregate through one-dimensional hydrogen bonding as well as its propensity for π–π stacking leads to highly anisotropic morphologies which can be linked to gel formation. Organogels are of particular interest as they have various applications in different areas like catalysis, biomedical research, drug delivery, and pharmaceutical crystallization [19,20,21,22,23,24,25,26,27]. For instance, there are numerous reports of designed-based gelators containing functionality like Bis(urea) [28,29]. Steed et al. have reported gelmorphs with different morphologies and different materials properties with acyl-semicarbazide moiety [30].

This work explores how solvent selection, based on solute–solvent interactions, affects the formation of supramolecular organogels of FmocFF. It examines the impact of solvents on organogel properties and potential gelmorphs, focusing on gel morphology and structure. Gelmorphs are essentially different gel states with varying structures and material properties that come from the same gelator [30]. It is important to fully understand gelmorphs to grasp the complexity of how they form from the same gelator, and to understand how the gel’s microstructure affects the properties of bulk materials. However, there are limited reports on multiple gel states as there are only a few studies [30]. The gel materials were thoroughly characterized by using spectroscopy, powder X-ray diffraction, and rheology techniques. We monitored the gelation process using scanning electron microscopy in the dried state and, furthermore, introduced in situ SHG microscopy as a novel technique to study gel structure in the native state. Moreover, FmocFF could be an excellent gelator for pharmaceutical crystallization, owing to its peripheral functional groups, which can interact with the API (active pharmaceutical ingredient) molecules and thus influence the crystallization outcome. The ability of FmocFF to form organogels in a wide range of organic solvents also supports its suitability as media for gel-mediated crystallization screening for pharmaceuticals.

## 2. Results and Discussion

FmocFF is highly effective as a hydrogel due to the presence of extensive hydrogen bond donors/acceptors, stabilizing the hydrogel. Gelation tests were conducted using 18 different solvents at varying concentrations (up to 2 wt%) (Table 1). The solvents were chosen based on their polarity and included aliphatic, aromatic protic, dipolar aprotic, and halogenated solvents. One criterion for the selection of a solvent is that it must dissolve FmocFF while enabling solute molecules to bind and form aggregates. The gelators were dissolved in 0.5 mL of the selected solvent, followed by 30–60 s of sonication until fully dissolved. The vials were left undisturbed at room temperature and checked for gelation at different intervals. A simple tube inversion test identified the gel formation. FmocFF has moderate solubility in organic solvents and is soluble upon heating, producing a gel upon cooling. Interestingly, solvents like methanol, 2-propanol, and γ-butyrolactone do not produce gels because of the higher solubility of FmocFF. However, gel formation can be observed at concentrations as low as 0.5 wt% in a 9:1 solvent–water ratio. FomcFF has formed translucent gels in nine different solvents (dichloromethane, chloroform, 1-butanol, 1-propanol, 2-butanol, octanol, acetonitrile, nitromethane, and toluene) at a concentration of 2 wt% (Figure S1). The critical gel concentration (CGC) for FmocFF is as low as 0.7 wt%. The CGC is 0.9 wt% in 1-propanol, 1.2 wt% in 1-butanol, and 1.9 wt% in 2-butanol. The CGC values are relatively high in alcoholic solvents. In contrast, the CGCs for halogenated solvents like DCM and chloroform are comparatively low (0.8 and 0.9 wt%, respectively), likely due to the low solubility of FmocFF. The CGC values tend to be low in non-alcoholic and non-polar solvents, likely due to a solvophobic effect caused by the highly lipophilic substituents and reinforced by strong self-association of the amide groups [15,31]. The hydrogen bond donating ability of solvent exhibited primary importance in controlling 1D self-assembly, and the hydrogen bond accepting ability and polarity of solvent mainly influenced the supramolecular interactions among assemblies and controlled the gels. Self-assembly favored a less polar solvent environment, potentially encouraging intramolecular hydrogen bonding between the carboxylic groups and groups’ C–H⋯π interaction at the phenyl rings [9,11]. In particular, based on the α parameter of solvents, the self-assembly in a given solvent can be predicted (Table S1). Alcoholic solvents with higher α parameters due to their hydrogen bonding competition with the gelator molecules produce weaker self-assembly, whereas solvents like DCM and chloroform with low α parameters will have minimum competition for hydrogen bonding. Therefore, the self-assembly of FmocFF molecules is expected to be stronger in non-polar solvents like toluene, with solvents like DCM, chloroform, and acetonitrile. 

**Figure 1:**
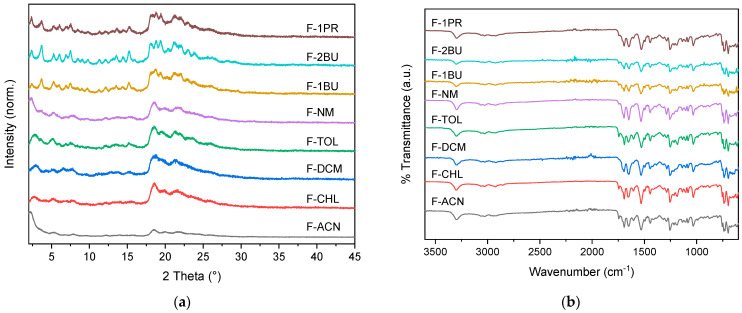
X-ray powder diffraction (**a**) and FTIR spectra (**b**) of FmocFF xerogels obtained from different solvents, in which xerogels prepared from 1-propanol, 1-butanol, 2-butanol, nitromethane, toluene, chloroform, dichloromethane, and acetonitrile are named as F-1PR, F-1BU, F-2BU, F-NM, F-TOL, F-CHL, F-DCM, and F-ACN, respectively.

X-ray powder diffraction (XRPD) was used to analyze xerogels obtained by the air-dried gel of the FmocFF organogels (Figure 1a). All the dried organogel samples exhibit low crystallinity. Comparatively more crystalline XRPD patterns are observed for xerogels obtained from alcoholic solvents, namely 1-propanol, 2-butanol, and 1-butanol. Structural differences between gels are evident from the characteristic peaks at 4, 6, and 15. XRPD patterns revealed that FmocFF rearranged and formed an orderly network structure during the self-assembly process for alcoholic solvents, driven by solute–solvent interactions [9]. The low-angle X-ray diffraction pattern of FmocFF xerogels in alcoholic solvents indeed exhibits periodic diffraction peaks, in line with an ordered supramolecular structure. For non-alcoholic solvents, the resulting XRPD patterns exhibit very low crystallinity. We therefore conclude that the selection of solvents influenced both the gelation behavior and overall crystallinity of the FmocFF organogels, reflecting the gelmorphic behavior of FmocFF. 

**Figure 2:**
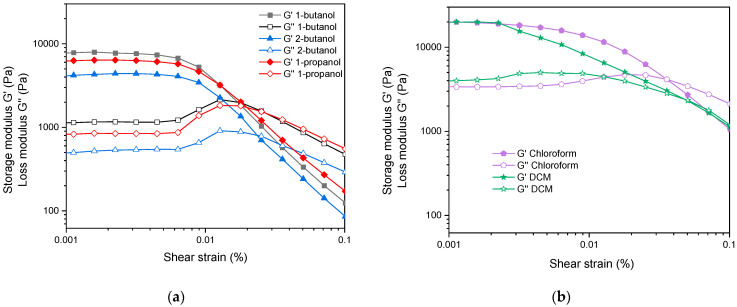
Rheology comparison of FmocFF gel obtained from alcoholic solvents (**a**) with gel obtained from chloroform and DCM (**b**).

The xerogels obtained from different solvents exhibited different FTIR spectra (Figure 1b). Significant band peak shifts occurred for the C=O from 1650 to 1648 cm^−1^ and for the NH stretching bands in the range from 3300 to 3306 cm^−1^. The shifts in -CONH signify the involvement of the amide groups in different H-bonding in the organogels. A shift in the C=O band suggests that the environment around the carboxyl group has changed, perhaps due to different conformations or interactions within the gel. Shifts in the aromatic stretching bands in the range 1527–1530 cm^−1^ suggest that the arrangement of the aromatic groups has been altered thereby indicating possible gel morphs. To relate the observed differences in gel structure to the mechanical properties a rheology experiment has been carried out to assess the mechanical stability of the gels. Rheology experiments were conducted at the same concentration (2 wt%) using different solvents (Figure 2a,b and Figure S2). Gels generally exhibited solid-like properties, with the storage modulus (G′) greater than the loss modulus (G″). Oscillatory rheology measurements confirmed the viscoelastic nature of the gel materials. Specifically, in all the gels, G′ was several orders of magnitude greater than G″, confirming the viscous nature of the FmocFF organogels. Yield stresses (σ) were determined for each gel to identify the point of breakdown, with higher yield stress values indicating greater gel stability. The rheological data clearly show that gels prepared from chloroform and DCM were highly robust, while those in alcoholic solvents had noticeably low σ values, indicating lower mechanical stability. For example, F-CHL and F-DCM exhibit yield stress (σ) values of 4153 Pa and 2337 Pa, respectively. In contrast, the organogels obtained from alcoholic solvents produced much weaker gels with yield stresses of around 703 Pa, 1810 Pa, and 1730 Pa in 2-butanol, 1-butanol, and 1-propanol, respectively. Moreover, a characteristic of weak strain overshoot behavior (type III) [32] has been observed for the gel obtained from alcoholic solvents which suggests that the structural breakdown is dependent on the strain, indicating lower-level energy storage associated with the formation of weak structural complexes within the organogels [33]. 

**Figure 3:**
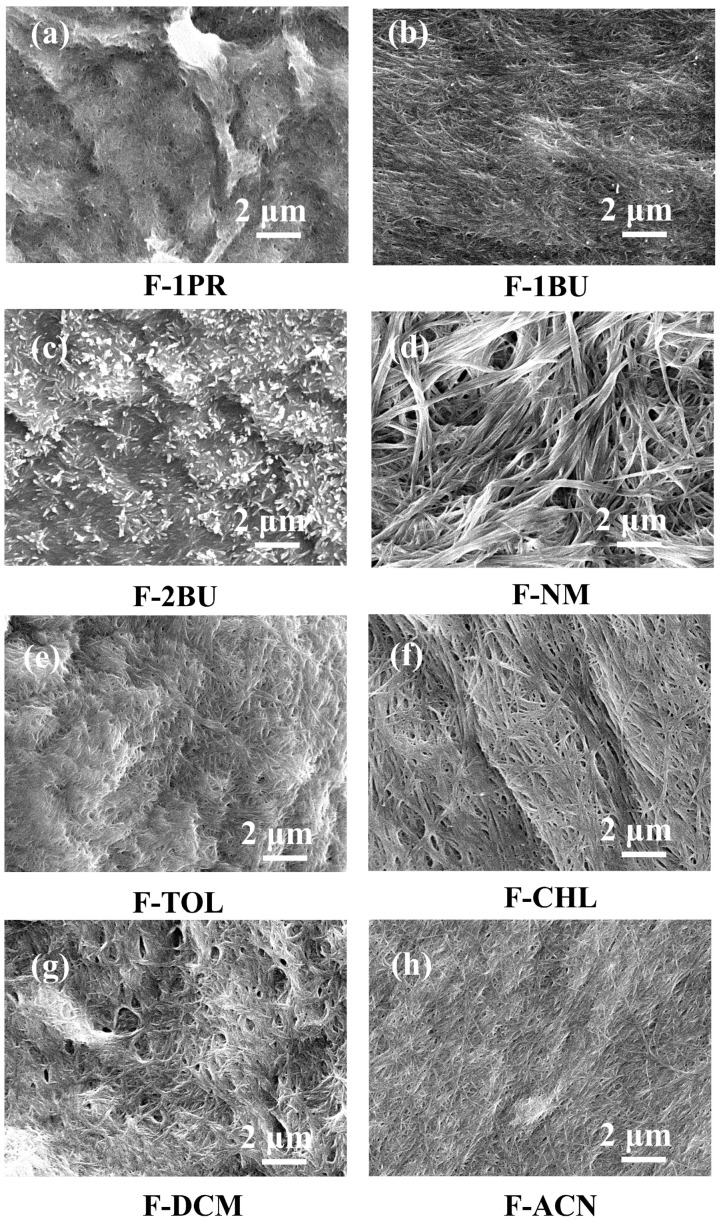
SEM micrographs of FmocFF xerogels prepared from 1-propanol (**a**, F-1PR), 1-butanol (**b**, F-1BU), 2-butanol (**c**, F-2BU), nitromethane (**d**, F-NM), toluene (**e**, F-TOL), chloroform (**f**, F-CHL), dichloromethane (**g**, F-DCM), and acetonitrile (**h**, F-ACN).

Scanning electron microscopy (SEM) was used to image the morphology of the FmocFF xerogels, revealing a highly knotted network in all samples (Figure 3). Due to differences in the self-assembly process across the solvents, the aggregators exhibited entirely distinct morphologies [30]. Particularly, clear differences in gel fibers are observed based on the gelation solvent. Analysis of organogels obtained from alcoholic solvents of air-dried gel shows that the xerogel of the FmocFF gel comprises thin fibril bunches. Locally, denser domains seem to form, most clearly in 2-butanol. In contrast, the dried organogel obtained in chlorinated solvents like dichloromethane and chloroform gives thicker fibrillar fibers (Figure 3). This observation confirms that the morphology of the xerogels is solvent system-dependent. Thus, these xerogels, arising from the same gelator, represent different gel structures that are tuned by their specific solute–solvent interaction.

While electron microscopy techniques are commonly used to study the nanoarchitecture of gels, it is important to consider that the sample preparation methods can affect the morphology of the gels. It is distinct from the SEM images above, where fibers in different samples tend to tangle with each other and extend in unnatural ways. Generally, the drying process during the xerogel preparation can lead to distortion to a certain degree due to mechanical forces interacting among fibers, which may not accurately represent the original morphology of the aggregating structures [34,35]. Moreover, SEM as such cannot directly provide information about crystalline order. To characterize gel structure in the native state, we applied second-harmonic-generation (SHG) microscopy to visualize the fibers in situ. SHG signals can be generated by simultaneous excitation with two photons, generating a single photon at twice the frequency of the input light. Like other second-order nonlinear optical processes, SHG is inherently sensitive to the structural order of materials, as it only occurs for noncentrosymmetric materials [36,37,38,39]. Some of us have exploited this sensitivity to gain structural insights into chiral crystals and liquid crystalline mesophases [40,41], to probe locally ordered domains within otherwise centrosymmetric crystalline phases [42], and to study aggregation in disordered polymer matrices [43]. In the case of FmocFF, we apply this methodology to evaluate the formation of ordered domains in the gel network. The (noncentrosymmetric) chiral supramolecular organization of FmocFF is expected to generate a detectable SHG signal. This signal will be most pronounced in highly ordered regions and absent in amorphous areas. In this manner, SHG microscopy allows the evaluation of the formation of ordered domains in the gel network. 

**Figure 4:**
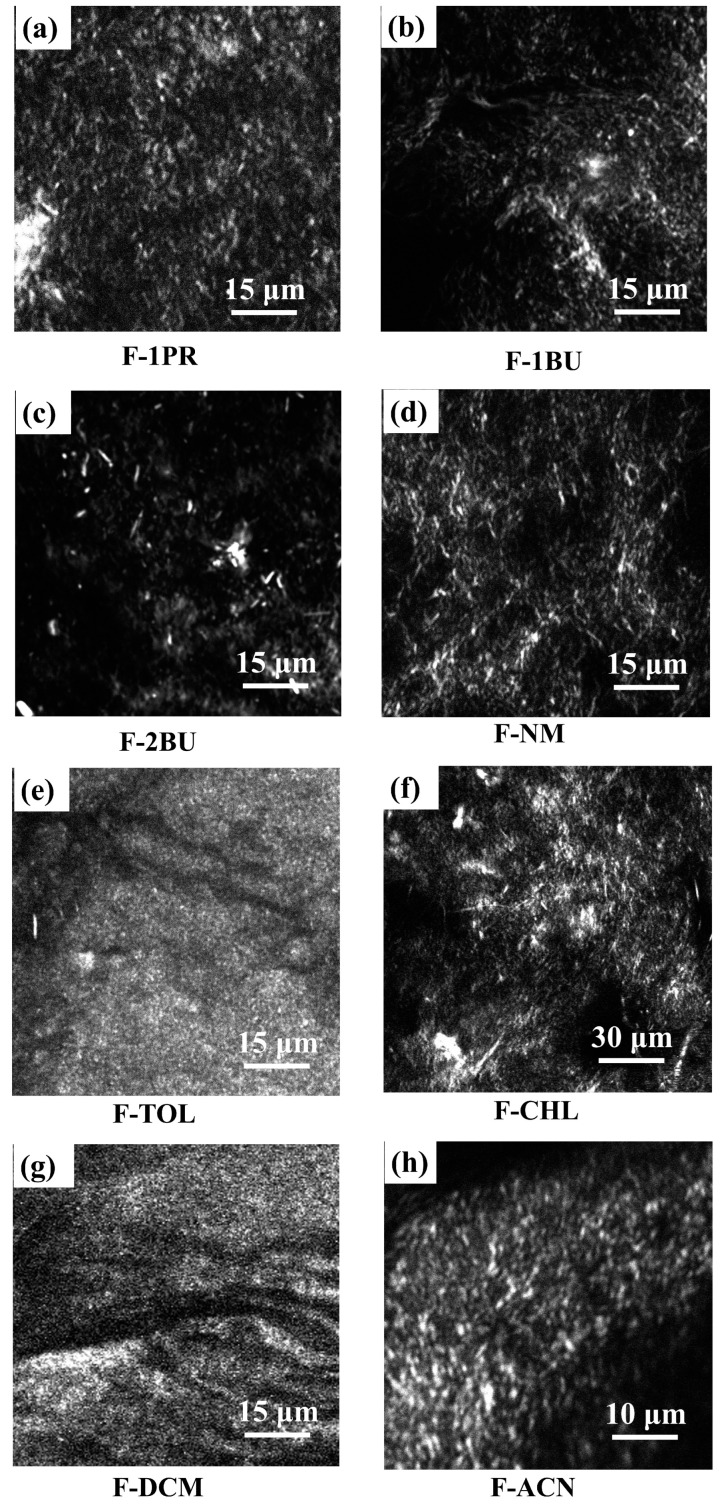
In situ SHG images of FmocFF organogels formed in 1-propanol (**a**, F-1PR), 1-butanol (**b**, F-1BU), 2-butanol (**c**, F-2BU), nitromethane (**d**, F-NM), toluene (**e**, F-TOL), chloroform (**f**, F-CHL), dichloromethane (**g**, F-DCM), and acetonitrile (**h**, F-ACN).

This structure-sensitive and label-free technique has been applied in the characterization of bio-tissues; however, it is rarely [44,45,46] used in supramolecular gel systems, although they share a lot of common physicochemical properties. Herein, we tested the SHG signals in situ for all organogels as prepared, to study the intrinsic structure of the gel fibrillar networks in the wet state, as shown in Figure 4. All organogels generate SHG, reflecting the long-range order of their fibrillar network. Compared with SEM, the SHG signals show consistent results; for example, thicker gel fibers in nitromethane-based gel also present a more distinguishable fibrillar network in its SHG image. For the alcoholic solvents, localized regions or domains showing a stronger SHG response than the surrounding matrix are observed. SHG is predominately observed in dense domains where molecular order or alignment is preserved over a large area. Therefore, areas with higher density and stronger structural order will dominate in the SHG images. This is particularly evident for 2-butanol, where ordered domains moreover present themselves as very regular needle-like crystallites. For 1-butanol, smaller domains of more irregular shape are apparent. For 1-propanol, large unstructured regions with strong SHG can be seen, indicating dense local domains with strong alignment. Note that in mixtures of organic solvents and water, ethanol–water did not lead to the formation of crystalline structures, whereas acetone–water mixtures promoted the formation of crystalline spherulites [15]. In acetonitrile, regions with higher SHG activity are also observed, but they are more evenly spread throughout the sample. For chloroform and nitromethane, long fibers are visible, sometimes extending over an order of tens of micrometers. Finally, for toluene and dichloromethane, a much more homogeneous SHG signal is observed overall. The observation of ordered domains in the alcoholic solvents, and particularly the highly regular crystallites in 2-butanol, seems in line with the XRPD observations pointing to higher crystallinity in the alcoholic organogels. The SHG microscopy experiments moreover show that these crystalline regions occur as distinct domains in a less organized gel network [47]. This can offer an explanation for the observed differences in mechanical stability between the alcoholic and non-alcoholic organogels, as the mechanical strength of a gel with a multi-domain structure relies on the strength of the links between the crystalline domains and the sample-spanning gel network [47]. We therefore assume that the lower mechanical stability of the alcoholic gels, as indicated by the strain overshoot in the rheometric measurements, is due to the individual ordered domains being physically separated and not strongly interconnected. This structural configuration results in weaker mechanical stability, even if crystallinity within the domains is high. This effect is the most pronounced for 2-butanol, where highly crystalline domains were observed through SHG microscopy, yet the organogel exhibited the lowest mechanical stability of all tested samples. Our findings of lower mechanical strength linked to crystallinity reflect the findings of Raeburn et al. in mixtures of water and organic solvent. In their study, they observed that acetone–water mixtures promoted the formation of crystalline spherulitic regions, which in turn led to reduced mechanical stability.

## 3. Conclusions

In conclusion, this study demonstrates the potential of FmocFF as an effective organogelator, capable of forming supramolecular gels across a broad spectrum of organic solvents. The gelation behavior, morphology, and mechanical properties of the resulting organogels were shown to be highly solvent-dependent, with variations in crystallinity and fibril architecture observed. Notably, solvents with lower polarity, such as dichloromethane and chloroform, yielded more mechanically stable gels compared to alcoholic solvents. The use of SHG microscopy provided insight into the in situ structure of the gels, and, in conjunction with XRPD data, led to the conclusion that the lower mechanical strength of alcoholic organogels is due to the formation of highly ordered domains that are only weakly connected to the gel network. One potential application of the FmocFF organogels can be as a crystallization medium for active pharmaceutical ingredients (APIs) to control crystal size, morphology, and polymorphic forms. Gel fibers serve as nucleation sites for crystallization, with the gelator’s functionality playing a significant role in the process. Finding a suitable gelator often relies on chance, and the search for a stable gelator for various APIs is ongoing. FmocFF is an excellent gelator due to its functional groups located at the periphery which can interact with API solutes, influencing crystallization outcomes. Moreover, an ongoing study shows it is possible to crystallize the same APIs in FmocFF using multiple organic solvents, highlighting its potential for crystallization screening.

## 4. Materials and Methods

### 4.1. Materials

Fmoc-Phe-Phe-OH (FmocFF, 95%) was purchased from abcr GmbH. Methanol (99%), dimethylformamide (DMF, 99%), dichloromethane (99%), chloroform (99%), ethyl acetate (99%), γ-butyrolactone (99%), cyclohexane (99%), 1,4-dioxane (99%), n-hexane (99%) and tetrahydrofuran (99%) were purchased from Fisher Scientific Company. In addition, 1-butanol (99%), 1-propanol (99%), 2-butanol (99%), 2-propanol (99%), octanol (99%), acetonitrile (99%), nitromethane (99%), and toluene (99%) were purchased from Sigma-Aldrich. All the reagents were used without further purification. Ultrapure water taken from the MILLI-Q Reference A+ System was used throughout the experiments.

### 4.2. Gel Preparation

For gel screening in pure organic solvents, an appropriate amount of FmocFF (up to 2 wt%) was dissolved in 0.5 mL of the selected organic solvent, followed by sonication at 40 °C until the solid fully dissolved. For gel screening in mixed solvents, a certain amount of gelator was dissolved in 0.5 mL solution with a 9:1 volume ratio of solvent to water. The vials were left undisturbed at room temperature and checked for gelation after cooling down. A simple tube inversion test identified the gel formation. For gel characterizations, all gel samples are prepared with a fixed gelator concentration at 2 wt%.

### 4.3. Characterizations

#### 4.3.1. Rheology

The rheological measurements were carried out using a modular compact rheometer (MCR 302e, Anton Paar, Graz, Austria) instrument. A plate–plate geometry of 10 mm diameter and a default gap of 1 mm was used, and a solvent trap kit was applied during the test. Strain sweeps were carried out between shear strains of 0.01% and 10%, with an angular frequency of 1 rad/s.

#### 4.3.2. Scanning Electron Microscopy (SEM)

Xerogel samples were prepared by air-drying process. For air drying, gels were left in ambient condition for natural drying for three days. All xerogel samples were then coated with platinum vapor (5 nm thickness) and analyzed on the scanning electron microscope (JSM-IT100, JEOL, Tokyo, Japan) operating at 10 kV.

#### 4.3.3. X-Ray Powder Diffraction (XRPD)

X-ray powder diffraction (XRPD) patterns were obtained on a Malvern PANalytical Empyrean diffractometer (Malvern, UK), equipped with a PIXcel3D solid-state detector using a Cu anode (Cu K_α1_: 1.5406 Å; Cu K_α2_: 1.5444 Å). The powders and gels dried at different conditions were loaded onto a 96-well sample holder and X-ray diffractograms were recorded at room temperature within a 2–45° 2θ range using a step size of 0.013°.

#### 4.3.4. Attenuated Total Reflectance–Fourier Transform Infrared (ATR–FTIR) Spectroscopy

An attenuated total reflectance–Fourier transform infrared (ATR–FTIR) spectrometer Bruker Alpha II (Billerica, USA) was utilized in this study, facilitating the mid-infrared (mid-IR) analysis of powder samples spanning the wavenumber range of 4000 to 400 cm^−1^. Each spectrum was generated with a spectral resolution of 4 cm^−1^, comprising 16 scan accumulations. To ensure uniformity, an adjustable knob applied consistent gauge pressure to powdered samples, and the diamond crystal underwent thorough cleaning with ethanol after each scanning cycle. Each experiment was repeated three times to guarantee repeatability. 

#### 4.3.5. Second Harmonic Generation Microscopy (SHG Microscopy)

For the SHG measurements, the microscope (BX61WI-FV1200-M, Olympus, Tokyo, Japan) was coupled to a mode-locked femtosecond laser (Insight DS+, Spectra-Physics, Santa Clara, CA, USA) emitting a vertically polarized beam with an average power output of 1.1 W at 1000 nm, a repetition rate of 80 MHz, and pulse durations of 120 fs. The entire setup was mounted on an anti-vibration optical table (TMC, USA) and enclosed in a custom-made Faraday cage constructed from aluminum and stainless steel. For all experiments, the laser excitation wavelength was set to 1000 nm. The output power was adjusted to 75 mW before entering the microscope by using a combination of a polarizer and an achromatic half-wave plate. At this power, sample degradation could be avoided. SHG images of the gel fibrillar framework were obtained with transmission mode using a single PMT. The filter setup included a narrow bandpass filter at 500 nm for SHG. A 40× water immersion long working distance objective (Nikon, NA = 0.8) is used in all measurements in combination with a visible light condenser (NA = 0.9). Before measurement, samples were sealed with a cover slip to avoid evaporation.

## Figures and Tables

**Table 1:** The gelation ability * of FmocFF with different organic solvents.

Solvents	Pure Solvent		Mixed with Water (φ_organic solvent_ ≈ 0.1)	
	Gelation Possibility	Concentration (mg/mL)	Gelation Possibility	Concentration (mg/mL)
Methanol	S	x	G	≥10
Acetonitrile	G	≥10	G	≥5
DMF	S	x	G	≥10
1-Propanol	G	≥9	G	≥10
2-Propanol	PG	≥20	G	≥5
1-Butanol	G	≥12	x	
2-Butanol	G	≥19	x	
Octanol	G	≥7	x	
Ethyl acetate	S	x	x	
γ-Butyrolactone	S	x	G	≥5
Toluene	G	≥10	x	
Dichloromethane	G	≥8	x	
Chloroform	G	≥9	x	
Cyclohexanone	S	x	x	
1,4-Dioxane	S	x	x	
n-Hexane	S	x	x	
Nitromethane	G	≥10	x	
Tetrahydrofuran	x		x	

* G = gel, PG = partial gel, S = soluble. Gel-forming systems are indicated in grey, with the corresponding critical gel concentration mentioned in the next column.

## Data Availability

All data and materials are available on request from the corresponding author.

## Supplementary Materials

The following supporting information can be downloaded at: https://www.mdpi.com/article/10.3390/gels10110749/s1, Figure S1: Optical image of the prepared organogels; Figure S2: Rheology FomocFF organogel obtained from (i) acetonitrile (ii) nitomethane and (iii) toluene; Table S1: Kamlet–Taft parameters of the eight selected solvents. α = hydrogen bond donor ability, β = hydrogen bond acceptor ability, and π* = polarizability. (source: https://www.stenutz.eu/chem/solv26.php, accessed on 14 November 2024).
