# Supplementary material for: Organogels of FmocFF: Exploring the Solvent-Dependent Gelmorphic Behavior
Source: Gels. 2024 Nov 19;10(11):749. doi: 10.3390/gels10110749 (PMC11594169; doi:10.3390/gels10110749)
Supplement: Supplementary file 1 [file gels-10-00749-s001.zip › gels-3288306-supplementary.pdf]

Supporting information

Table S1: Kamlet–Taft parameters of the eight selected solvents. α = hydrogen bond donor ability, β = hydrogen bond acceptor ability, and π* = polarizability. (source: https://www.stenutz.eu/chem/solv26.php)

**Solvent**	**α (hydrogen bond donor)**	**β (hydrogen bond acceptor)**	**π* (polarizability)**
1-butanol	0.84	0.84	0.47
2-butanol	0.69	0.80	0.40
1-propanol	0.84	0.90	0.52
Chloroform	0.44	-	-
Dichloromethane	0.30	-	-
Acetonitrile	0.19	0.40	0.75
Toluene	0.00	0.11	0.54
Nitromethane	0.22	0.06	0.85

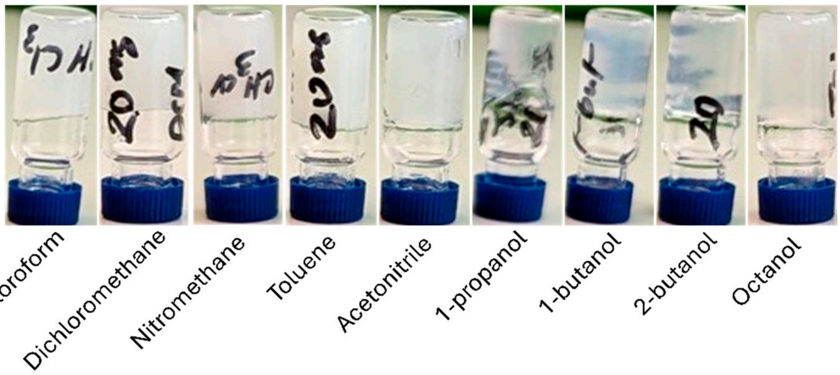

Figure S1: Optical image of the prepared organogels.

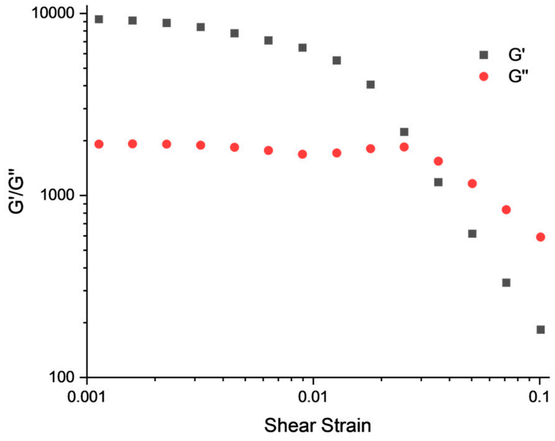

(i)

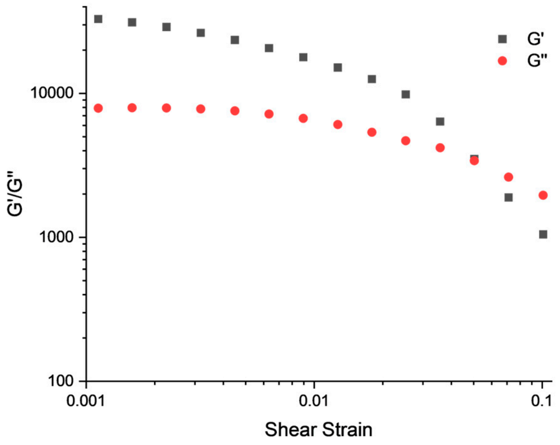

(ii)

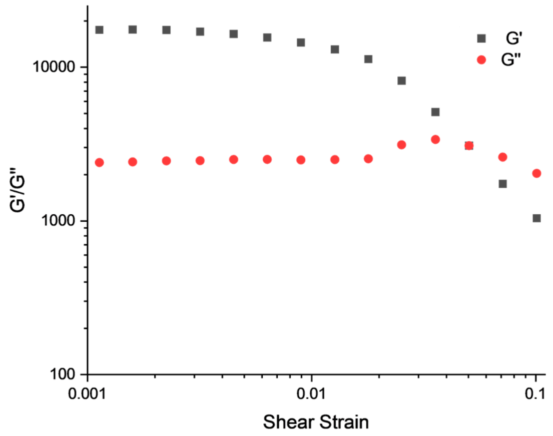

(iii)

Figure S2: Rheology FomocFF organogel obtained from (i) acetonitrile (ii) nitomethane and (iii) toluene.
